# Nuclear Receptor-Targeted Therapy for Metabolic Dysfunction-Associated Steatotic Liver Disease

**DOI:** 10.53941/ijddp.2025.100024

**Published:** 2025-12-15

**Authors:** Yachao Zhou, Zhaojian Liu, Donghai Cui, Xingyun Qi, Huiliang Zhang

**Affiliations:** 1 Department of Medical Genetics and Molecular Biochemistry, Lewis Katz School of Medicine, Temple University, Philadelphia, PA 19140, USA; 2 School of Basic Medical Sciences, Shandong University, Ji’nan 250012, China; 3 Department of Biology, Rutgers University, Camden, NJ 08102, USA

**Keywords:** metabolic dysfunction-associated steatotic liver disease (MASLD), nuclear receptors (NRs), thyroid hormone receptor-β (THR-β), peroxisome proliferator-activated receptors (PPARs), farnesoid X receptor (FXR), nuclear receptor targeted therapy

## Abstract

Metabolic dysfunction-associated steatotic liver disease (MASLD) is the most prevalent chronic liver disease worldwide, affecting over 25% of the global population. Metabolic dysfunction-associated steatohepatitis (MASH) is an advanced stage of MASLD, characterized by hepatic steatosis accompanied by inflammation, hepatocellular injury, and fibrosis. Despite its high prevalence and clinical significance, effective treatments for MASLD and MASH remain limited, largely due to the complexity of the underlying pathophysiological mechanism, which remains not yet fully understood. Nuclear receptors (NRs) are a superfamily of transcription factors and play a key role in regulating lipid metabolism, glucose homeostasis, inflammation, and fibrosis, all of which are central to MASLD progression. Consequently, NRs have emerged as promising molecular targets for MASLD treatment, and a few new NR-targeted drugs were approved recently, including thyroid hormone receptor-β (THR-β) agonist resmetirom, the dual peroxisome proliferator-activated receptor (PPAR)-α/γ agonist saroglitazar. Moreover, several NR-targeted drugs are under clinical trials. In this mini-review, we summarize the recent progress of the mechanisms of key NRs in the pathogenesis of MASLD, and discuss the advances in nuclear receptor-targeted therapy, with emphasis on THR-β, PPARs, and the non-bile acid farnesoid X receptor (FXR).

## 1. Introduction

Metabolic dysfunction-associated steatotic liver disease (MASLD), previously known as nonalcoholic fatty liver disease (NAFLD), is a hepatic manifestation of metabolic syndrome strongly associated with obesity and insulin resistance [1]. The simple hepatic steatosis is asymptomatic, however, it can progress to metabolic dysfunction-associated steatohepatitis (MASH), a more severe stage of MASLD. MASH is characterized by inflammation, hepatocellular injury, and fibrosis, which can progress to steatohepatitis, cirrhosis, and hepatocellular carcinoma [2,3]. With a global prevalence exceeding 25%, MASLD is the most common liver disease among children and adolescents in developed countries [4–7], and its incidence continues to rise in parallel with increasing rates of obesity [8]. Mortality related to MASLD-associated liver complications is projected to more than double between 2016 and 2030 [9]. The MASLD/MASH diseases exhibit substantial phenotypic heterogeneity, and to date, no effective pharmacological therapies are available. Advanced stages of MASLD/MASH can ultimately result in liver failure, making it the second leading indication for liver transplantation [10].

Nuclear receptors (NRs) are a superfamily of ligand-regulated transcription factors that respond to small lipophilic ligands. In humans, 48 NRs have been identified, each characterized by conserved structural domains, including a N-terminal ligand-independent transcriptional activation domain (AF-1), a DNA-binding domain (DBD), a ligand-binding domains (LBDs) located within the hinge region which extends into the C-terminus, and another ligand-independent transcriptional activation domain (AF-2) [11]. Endogenous ligands for NRs include steroid and thyroid hormones, retinoids, and metabolites of vitamins, fatty acids, and cholesterol [12]. Upon ligand binding, NRs undergo conformational changes and are activated, enabling their translocation to the nucleus. In their basal state, some NRs reside in the nucleus and are pre-associated with corepressor proteins, which maintain them in an inactive conformation [13]. Once activated, they bind to specific DNA sequences, known as regulatory elements, in the promoter regions of target genes, where they recruit co-regulators to modulate transcriptional activity, either enhancing or repressing gene expression [14]. NRs play central roles in regulating key metabolic and physiological processes, including hepatic lipid and glucose metabolism, energy homeostasis, bile acid (BA) regulation, inflammation, fibrosis, and cellular proliferation. Agonists targeting specific NRs have shown therapeutic potential and are being actively investigated as treatments for MASLD [15].

The thyroid hormone receptor-β (THR-β), peroxisome proliferator-activated receptors (PPARs), and farnesoid X receptor (FXR) are among the most extensively studied NRs as therapeutic targets for liver diseases. Rezdiffra, a selective THR-β agonist, is the first agent in this class approved by the U.S. Food and Drug Administration (FDA) for the treatment of MASH in patients with moderate to advanced fibrosis [16]. Clinical trials have demonstrated its efficacy in inducing histological resolution of MASH without exacerbating fibrosis, marking a significant advancement in NR-targeted therapies for MASLD. PPAR-γ agonists, such as rosiglitazone and pioglitazone, have been shown to improve insulin sensitivity and modulate glucose and lipid metabolism in individuals with type 2 diabetes mellitus, and have also been employed in the prevention and treatment of MASLD [17,18]. Notably, activation of PPAR-α and PPAR-β/δ has also demonstrated positive effects on the treatment of MASH [19,20]. Furthermore, dual and pan-PPAR agonists exhibit enhanced therapeutic potential by targeting multiple metabolic pathways simultaneously [21]. In 2020, saroglitazar, a dual PPAR-α/γ agonist, received regulatory approval from the Drugs Controller General of India for the treatment of MASH [22]. FXR, a pivotal regulator of bile acid (BA) synthesis and metabolic signaling, is another promising target. Obeticholic acid (OCA), a potent FXR agonist, has been shown to significantly improve insulin resistance and reduce hepatic inflammation and fibrosis markers in patients with MASLD [23].

In this mini-review, we provide an overview of the role of NRs in the pathophysiology of MASLD, with a particular focus on their regulatory functions in metabolic pathways. Additionally, we highlight recent advances in NR-targeted therapeutic strategies, emphasizing key receptors such as THR-β, PPARs, and FXR, along with their corresponding agonists currently in development or approved.

## 2. Roles of Nuclear Receptors in MASLD Pathogenesis and Metabolic Regulation

As a central metabolic organ, the liver plays a vital role in maintaining systemic metabolic homeostasis through a complex and tightly regulated network of biochemical signals and cellular pathways, particularly those involved in glucose and lipid metabolism. In glucose homeostasis, the liver orchestrates essential processes such as glycolysis, gluconeogenesis, glycogenolysis, and glycogen synthesis [24]. In lipid metabolism, it regulates lipid uptake, de novo lipid synthesis, fatty acid β-oxidation, and secretion of very-low-density lipoproteins (VLDLs), thereby maintaining lipid balance [25]. The pathogenesis of MASLD is multifactorial, driven by insulin resistance, hormonal imbalances, dietary factors, alterations in gut microbiota, and genetic and epigenetic influences. These factors collectively disrupt hepatic metabolic equilibrium, promote oxidative stress, and trigger pro-inflammatory signaling cascades, contributing to disease initiation and progression [26,27]. NRs are key regulators of hepatic physiology, governing not only lipid metabolism but also integrating multiple pathways critical for MASLD pathogenesis, including lipid accumulation, insulin resistance, mitochondrial dysfunction, endoplasmic reticulum stress, and fibrogenesis [15].

### 2.1. Key Nuclear Receptors and Transcriptional Regulation in MASLD

#### 2.1.1. THR-β: A Key Regulator of Hepatic Lipid Oxidation and MASH Therapy

**Figure 1.**
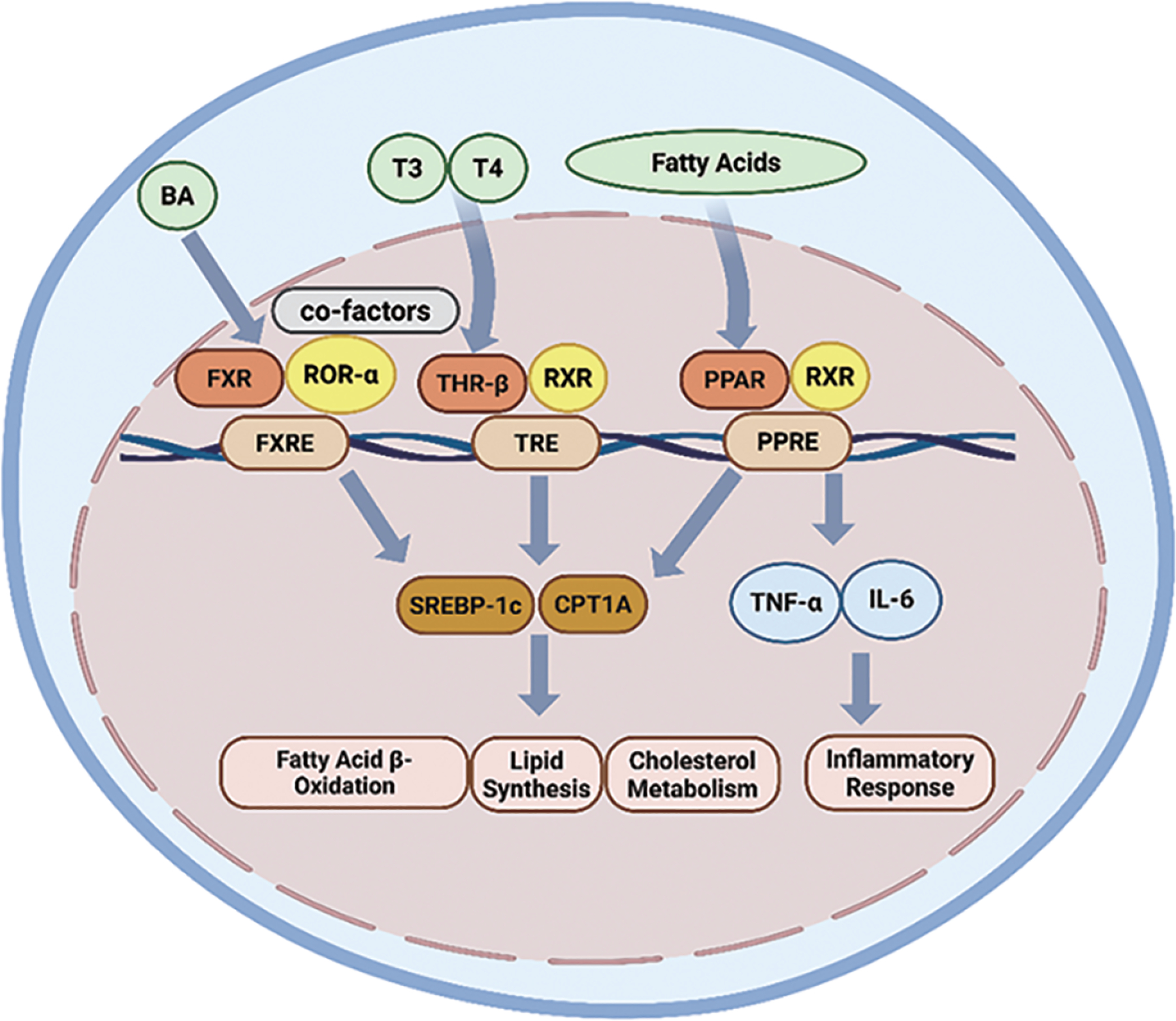
The impact of nuclear receptors on liver metabolism and inflammatory pathways. Nuclear receptors, including FXR, THR-β, and the PPAR family, bind to their ligands and influence the expression of proteins involved in cellular glycolipid metabolism (SREBP-1c, CPT1A) and cellular immune pathways (TNF-α, IL-6). BA: bile acid; RORα: retinoic acid-related orphan receptor α; FXRE: FXR response element; TRE: thyroid response element; RXR: retinoid X receptors; PPRE: PPAR-γ response element. This activation subsequently affects hepatic fatty acid β-oxidation, lipid synthesis, cholesterol metabolism, and immune responses.

NRs are expressed in the liver, where they regulate key metabolic processes through transcriptional control mechanisms [15] (Figure 1). Among them, the thyroid hormone receptor (THR) exists in two main isoforms, THR-α and THR-β, which exhibit tissue-specific expression patterns. THR-β is the predominant isoform in hepatic tissue and plays a central role in liver metabolism; however, its expression is notably diminished in patients with MASH [28]. The human THR-β (NR1A2) gene, located on chromosome 3, encodes THR-β comprising 461 amino acids.

The hypothalamus initiates the thyroid hormone axis by secreting thyrotropin-releasing hormone (TRH), which stimulates the anterior pituitary gland to release thyroid-stimulating hormone (TSH) [29]. In turn, the thyroid gland secretes thyroxine (T4) and its biologically active form, triiodothyronine (T3). T3 binds to THR-β to regulate the transcription of target genes [30]. Activation of THR-β influences a wide range of biological processes relevant to the pathogenesis of MASLD. It suppresses hepatic lipogenesis by inhibiting the expression of the lipogenic transcription factor sterol regulatory element-binding protein 1c (SREBP-1c), thereby reducing de novo lipid synthesis [31]. Also, THR-β activation promotes triglycerides (TGs) hydrolysis by enhancing the expression and activity of hepatic lipase, facilitating the breakdown of TGs into glycerol and fatty acids within hepatocytes [32]. Furthermore, THR-β increases mitochondrial biogenesis and promotes the β-oxidation of fatty acids by activation of carnitine palmitoyltransferase 1A (CPT1A), pyruvate dehydrogenase kinase 4 (PDK4), and uncoupling protein 2 (UCP2) [33].

#### 2.1.2. PPARs: Multifaceted Regulators of Fatty Acid Metabolism and Inflammation

PPARs are critical regulators of metabolic diseases, with key roles in lipid and glucose metabolism, energy homeostasis, and modulation of inflammation and fibrosis [34]. The PPAR subfamily comprises three isoforms: PPAR-α, PPAR-β/δ, and PPAR-γ. These PPARs are activated by endogenous ligands, including fatty acids, arachidonic acid derivatives, and oxidized phospholipids. Upon ligand binding, the PPARs heterodimerize with the retinoid X receptor (RXR) and bind to specific peroxisome proliferator response elements (PPREs) in the promoter regions of target genes to initiate transcription [21].

The human PPAR-α (NR1C1) gene is located on chromosome 22 and encodes the PPAR-α comprising 468 amino acids. PPAR-α is primarily expressed in metabolically active tissues such as liver, skeletal muscle, brown adipose tissue, and the heart [35]. PPAR-α upregulates the expression of genes regulating fatty acid metabolism and mitochondrial β-oxidation. Key targets of PPAR-α include CPT1A, carnitine palmitoyltransferase 2 (CPT2), and medium-chain acyl-CoA dehydrogenase (ACADM), which collectively promote the breakdown of fatty acids to generate energy [19]. In addition, PPAR-α agonists have been used to reduce coronary artery disease by lowering TG levels and increasing high-density lipoprotein (HDL), without significantly affecting low-density lipoprotein (LDL) levels [34].

The human PPAR-β/δ (NR1C2) gene is located on chromosome 6 and encodes a protein with 441 amino acids. PPAR-β/δ is predominantly expressed in hepatocytes, Kupffer cells, sinusoidal endothelial cells, and hepatic stellate cells (HSC) [36]. PPAR-β/δ regulates multiple metabolic pathways, in part through activation of adenosine monophosphate-activated protein kinase (AMPK) [37]. This includes inhibition of lipogenesis, suppression of glycogen synthesis, reduction of gluconeogenesis, and enhancement of fatty acid oxidation [20]. Additionally, PPAR-β/δ suppresses lipogenesis by downregulating the expression of SREBP-1c, a key transcription factor in lipid synthesis [20]. Agonists targeting PPAR-β/δ are believed to enhance lipid catabolism, thereby reducing lipid accumulation associated with metabolic syndrome and insulin resistance [38].

The human PPAR-γ (NR1C3) gene is located on chromosome 3 and is predominantly expressed in adipose tissue and the immune system [35]. PPAR-γ encodes two major isoforms: PPAR-γ1, comprising 477 amino acids, and PPAR-γ2, comprising 505 amino acids. PPAR-γ plays a pivotal role in adipocyte differentiation, adipogenesis, and lipid metabolism, while also contributing to the improvement of insulin resistance, inflammation, oxidative stress, endoplasmic reticulum (ER) stress, and fibrosis [21]. In adipose tissue, PPAR-γ upregulates the expression of genes such as lipoprotein lipase (LPL), fatty acid-binding protein 4 (FABP4), and fatty acid synthase (FAS), facilitating the uptake and storage of circulating lipids [21]. In hepatocytes, PPAR-γ similarly promotes lipogenesis by increasing the expression of SREBP-1c, FAS, and lipoprotein lipase (LPL) [39]. Additionally, it enhances fatty acid uptake and hepatic lipid accumulation by upregulating the expression of cluster of differentiation 36 (CD36) and fatty acid transport protein (FATP) [21]. Notably, hepatic expression of PPAR-γ is significantly elevated in individuals with MASLD [40].

PPAR-γ also exerts antioxidant effects by suppressing the production of reactive oxygen species (ROS) through inhibition of inducible nitric oxide synthase (iNOS) and by activating protective factors such as nuclear factor erythroid 2-related factor 2 (NRF2) and secreted frizzled-related protein 5 (SFRP5) [41]. Furthermore, PPAR-γ alleviates endoplasmic reticulum (ER) stress by downregulating ER stress-related proteins, including activating transcription factor 4 (ATF4), C/EBP homologous protein (CHOP), and eukaryotic initiation factor 2α (eIF2α) [42]. Lastly, PPAR-γ mitigates hepatic inflammation by transcriptionally repressing pro-inflammatory transcription factors such as nuclear factor kappa-B (NF-κB), activator protein 1 (AP-1), and signal transducer and activator of transcription 1 (STAT1) [43].

PPARs also regulate metabolism through fibroblast growth factor 21 (FGF21), a hepatokine activated by PPAR-α and PPAR-γ in the liver [19]. The activated FGF21 enhances insulin sensitivity, promotes mitochondrial β-oxidation in skeletal muscle, and suppresses hepatic lipogenesis by inhibiting SREBP-1c [44].

#### 2.1.3. Farnesoid X receptor (FXR): Bile Acid Homeostasis and Systemic Metabolic Control

FXR is a key nuclear receptor that regulates bile acid (BA) synthesis, and is primarily expressed in the liver and intestine [45]. The human FXR (NR1H4) gene is located on chromosome 12 and encodes the FXR 447 amino acids. Upon activation, FXR forms a heterodimeric complex with retinoid X receptor (RXR), undergoes a conformational change, exposes the activation function-2 (AF-2) domain, and recruits coactivator proteins to regulate the transcription of target genes [46].

FXR functions as a central enterohepatic regulator, maintaining BA homeostasis and modulating lipid and glucose metabolism, insulin sensitivity, and inflammation [47]. One of its primary actions is to induce the expression of Small Heterodimer Partner (SHP) by directly binding to its promoter [46]. The activated SHP, in turn, inhibits the activity of SREBP-1c, thereby reducing fatty acids synthesis [46]. Additionally, FXR enhances insulin sensitivity through activation of the phosphatidylinositol 3-kinase (PI3K)/protein kinase B (PKB, as known as AKT)/glycogen synthase kinase 3 (GSK3) signaling cascade. This is achieved by increasing glycogen content and promoting the translocation of glucose transporter 2 (GLUT2) [48]. Activation of FXR also promotes the secretion of fibroblast growth factor 15 (FGF15, in murine) and FGF19 (in human), which circulate to the liver via the portal vein and bind to hepatic fibroblast growth factor receptor 4 (FGFR4) [49]. This axis inhibits cholesterol 7α-hydroxylase (CYP7A1), reducing bile acid synthesis and redirecting cholesterol toward neutral lipid storage. Concurrently, FGF15/19 enhances hepatic fatty acid oxidation by activating adenosine monophosphate-activated protein kinase (AMPK) [50].

### 2.2. Role and Mechanisms of Key Nuclear Receptors in the Pathogenesis of MASLD

**Figure 2.**
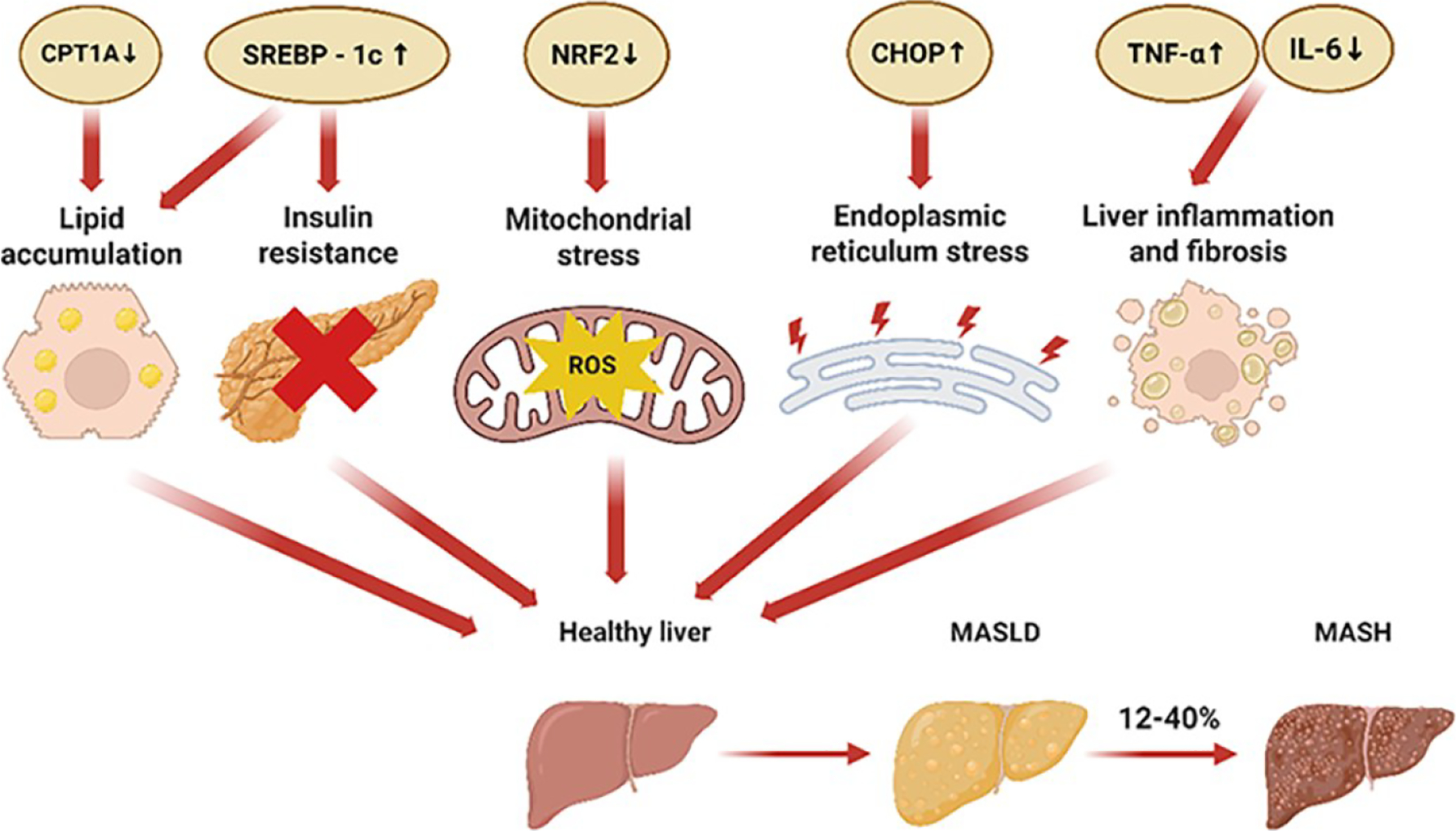
The nuclear receptors activate signaling proteins in the pathophysiology of MASLD. The nuclear receptors activate SREBP-1c, CPT1A, NRF2, CHOP, TNF-α, and IL-6 involved in the pathogenesis of MASLD. The healthy liver undergoes hepatic lipid accumulation, insulin resistance, mitochondrial stress, endoplasmic reticulum stress, and fibrotic response to develop MASLD. Without timely intervention, MASLD progresses to MASH, marked by inflammation and hepatocellular injury.

The progression of MASLD primarily involves a cascade of interrelated pathological events, including hepatic lipid accumulation, insulin resistance, mitochondrial dysfunction, endoplasmic reticulum (ER) stress, and activation of the fibrosis response [15]. Importantly, these factors do not function in isolation; rather, they are highly interconnected and mutually reinforcing, collectively driving the onset and progression of MASLD (Figure 2). Following, we will explore the molecular mechanisms by which nuclear receptors modulate the MASLD pathological processes.

#### 2.2.1. Lipid Accumulation

One of the key hallmarks of MASLD is the accumulation of intrahepatic triglycerides (TGs) [51]. Although the liver is not a primary site for lipid storage, ectopic fat deposition can occur under pathological conditions. Factors such as obesity, insulin resistance, and metabolic syndrome result in an excess of fatty acids in the liver. This lipid overload disrupts normal hepatic metabolic processes, impairs hepatocellular function, and promotes the development of MASLD [52].

The hepatic lipid is derived from three primary sources. (1) De novo lipogenesis (DNL): Although DNL contributes only approximately 5% of total hepatic lipid content [53], it is a critical driver of MASLD pathogenesis [54,55]. DNL is primarily regulated by the transcription factors SREBP-1c and carbohydrate response element binding protein (ChREBP), both of which stimulate DNL when they are activated [54]. Notably, nuclear receptors modulate both SREBP-1c and ChREBP. For example, PPAR-β/δ activation suppresses SREBP-1c expression, thereby attenuating hepatic steatosis [36]. (2) Postprandial lipid uptake: Following food intake, lipids absorbed from the intestine are transported to the liver via the hepatic portal vein. This process involves the uptake of circulating lipid-rich particles and is primarily mediated by the transport proteins FATP and CD36, and PPAR-γ enhances the expression of both FATP and CD36, thereby promoting hepatic lipid uptake during the fed state [21]. (3) Adipose tissue lipolysis (during fasting): In the fasting state, adipocytes mobilize stored TGs, releasing free fatty acids (FFAs) into the circulation. These FFAs are subsequently taken up by liver through the action of CD36, FATP, and PPAR-γ [21]. This is a physiological response to energy demand during fasting. However, the chronic elevation of circulating FFAs in obesity and insulin resistance can lead to excessive hepatic lipid accumulation and contribute to MASLD progression.

The fate of hepatic lipids is determined by the balance between lipid storage, oxidation, and export. The two principal metabolic pathways that regulate hepatic lipid disposal include: (1) Lipid secretion via very low-density lipoprotein (VLDL): The liver exports TGs into the blood primarily through the assembly and secretion of VLDL particles [56]. This process is tightly regulated by a network of metabolic factors, including ChREBP, carnitine palmitoyltransferase (CPT), fatty acid binding protein (FABP), PPAR-α, and SREBP-1c [57]. In individuals with MASLD, PPAR-α expression is downregulated, resulting in reduced hepatic lipid export and contributing to intrahepatic lipid accumulation [58]. (2) Fatty acid β-oxidation for energy production: β-oxidation is the principal catabolic pathway for fatty acids, occurring primarily in the mitochondria and, to a lesser extent, in peroxisomes [59]. PPAR-α activation upregulates the transcription of genes involved in β-oxidation, including the CPT1A and the medium chain-acyl-CoA dehydrogenase (MCAD), thereby facilitating lipid catabolism and reducing hepatic lipid levels [60]. However, when TGs production in the liver exceeds the combined capacity for VLDL secretion and β-oxidation, excess lipids accumulate within hepatocytes, contributing to hepatic steatosis and progression of MASLD [61].

#### 2.2.2. Insulin Resistance

Insulin resistance arises when tissues exhibit a diminished response to insulin, resulting in impaired glucose uptake and hyperglycemia. To compensate, the pancreas increases insulin production and secretion to maintain normal blood glucose levels [62]. Persistent hyperglycemia promotes de novo lipogenesis, contributing to hepatic lipid accumulation and accelerating the progression of MASLD [52]. Mechanistically, PPAR-γ enhances insulin sensitivity by upregulating the transcription of phosphatidylinositol 3-kinase (PI3K) and glucose transporter type 4 (GLUT4), both of which are critical components in the insulin signaling pathway [63].

#### 2.2.3. Mitochondrial Dysfunction

The liver is among the organs with the highest mitochondrial density. Mitochondria are essential for a broad spectrum of hepatic functions, ranging from substrate metabolism, energy production, cellular signaling, to the biotransformation of exogenous substances [64]. Excessive lipid accumulation in hepatocytes elevates the production of reactive oxygen species (ROS), a class of highly reactive oxidative molecules that includes superoxide anions, hydroxyl radicals, and hydrogen peroxide [65]. The excessive ROS induce oxidative damage to polyunsaturated fatty acids (PUFAs) in the mitochondrial membrane and impair the proper assembly of the mitochondrial respiratory chain complexes, leading to mitochondrial dysfunction and further aggravating hepatic steatosis [66]. Activation of PPAR-γ by agonists has been shown to upregulate the expression of NRF2 and SFRP5, thereby reducing ROS levels, mitigating oxidative stress and inflammation, and ultimately slowing the progression of MASLD [41].

#### 2.2.4. Endoplasmic Reticulum (ER) Stress

ER stress promotes the progression of hepatic steatosis from simple steatosis to MASH. Under conditions of ER stress, the folding of nascent peptides and proteins is impaired, as this process is normally governed by ER-resident enzymes and chaperones [67]. Improperly folded or unfolded proteins accumulated within the ER lumen, triggering the unfolded protein response (UPR). The UPR disrupts ER homeostasis and activates ER stress-related genes, including growth arrest and DNA damage inducible gene 153 (Gadd153) and ATF4 [67]. Prolonged ER stress can activate various intracellular stress pathways, thereby exacerbating insulin resistance, inflammation, and autophagy abnormalities, all of which contribute to the pathogenesis of MASLD/MASH [68]. Pharmacological activation of FXR by compounds, such as betulinic acid, attenuates ER stress and downregulates UPR markers, offering potential therapeutic benefits [69].

#### 2.2.5. Fibrosis Response

Although hepatic steatosis may initially present as a benign condition, it can progress to hepatocellular injury and inflammation, thereby initiating immune activation. Early lipid accumulation in hepatocytes triggers ER stress and mitochondrial dysfunction, leading to the release of damage-associated molecular patterns (DAMPs), which activate Kupffer cells and recruit infiltrating macrophages to the liver [70]. In response, these immune cells secrete pro-inflammatory cytokines, such as interleukin-6 (IL-6), interleukin-1β (IL-1β), and tumor necrosis factor-α (TNF-α), through nuclear factor-κB (NF-κB)-mediated transcription [71]. Concurrently, hepatic stellate cells (HSCs) undergo trans differentiation into myofibroblast-like cells under the influence of profibrotic factors such as transforming growth factor-β (TGF-β) and platelet-derived growth factor (PDGF) [72]. This transition leads to the excessive deposition of extracellular matrix components, including collagen types I/III, fibronectin, and laminin [72]. PPAR-γ inhibits the expression of key fibrogenic markers such as connective tissue growth factor (CTGF) and α-smooth muscle actin (α-SMA), thereby suppressing the activation of HSCs [73]. Moreover, both PPAR-γ and FXR mitigate hepatic inflammation by inhibiting the expression of inflammatory factors through suppression of NF-κB transcription [43,74].

The multifaceted roles of NRs in regulating the progression of MASLD form the basis for the development of NR-targeted therapeutic strategies. These mechanistic insights have already been translated into clinical applications, with several NR-modulating agents advancing to clinical trials or receiving regulatory approval, underscoring the therapeutic potential of targeting NR signaling pathways. See detailed discussion in Section 3.

## 3. Nuclear Receptor-Targeted Therapy for MASLD

Current management strategies for MASLD encompass non-pharmacological interventions, non-targeted pharmacological therapies, and drug-targeted treatments. Non-pharmacological strategies, such as dietary modifications, exercise interventions, and bariatric surgery, are the fundamental management of MASLD and are considered essential in all stages of the disease treatment [75]. Notably, the National Institute for Health and Care Excellence (NICE) guidelines recommend lifestyle changes as the primary treatment for MASLD [76]. However, the effectiveness of these interventions is limited in the end stages of MASLD, particularly in MASH.

Non-targeted pharmacological therapies mainly include antioxidants and hepatoprotective drugs, which help slow the progression of MASLD but do not target a specific pathway [75]. In contrast, drug-targeted therapies are designed to interfere with specific molecular pathways implicated in MASLD. These therapies focus on four main mechanisms. (1) Hepatic lipid metabolism, targeting effector molecules involved in the fat accumulation, such as nuclear receptor agonists, inhibitors of adipogenesis, and FGF21 analogues [77]. (2) Inflammation and apoptosis, such as kinase inhibitors of apoptotic and inflammatory signaling pathways [78]. (3) Gut-liver axis dysregulation, including strategies aimed at modulating the gut microbiome and reducing metabolic endotoxemia [79]. (4) Fibrosis progression, by inhibiting signaling molecules and pathways that drive hepatic fibrogenesis [78].

**Figure 3.**
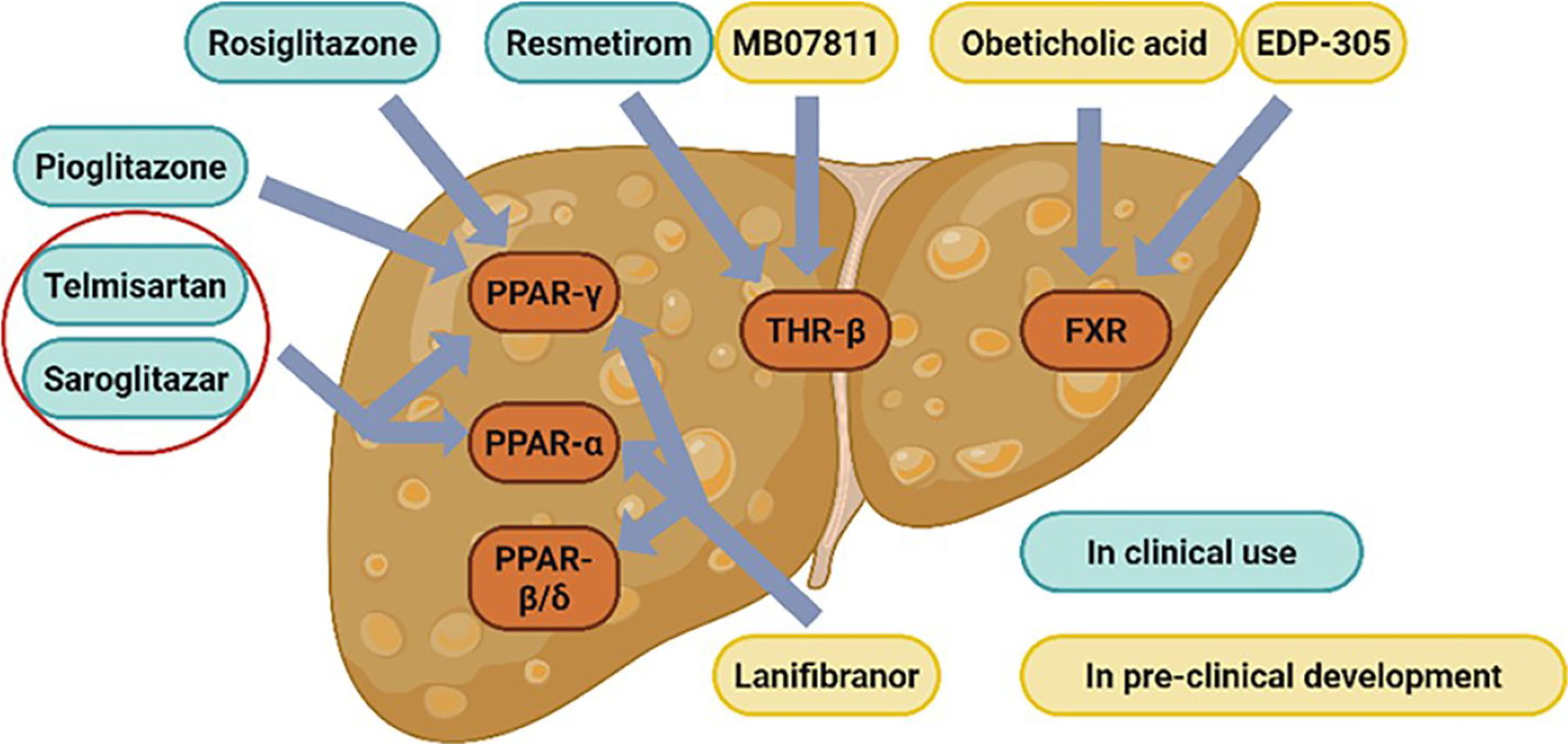
Nuclear receptor-targeted therapies for MASLD. This figure depicts the targets and clinical development stages of nuclear receptor-targeted drugs for MASLD. It highlights compounds in pre-clinical development (yellow: MB07811, Lanifibranor, Obeticholic acid, EDP-305) as well as those in clinical use (blue: Resmetirom, Rosiglitazone, Pioglitazone, Telmisartan, Saroglitazar). Telmisartan and Saroglitazar are dual PPAR-α/γ agonists.

Given the extensive network of metabolic pathways regulated by NRs, modulation of a single NR can elicit a wide range of biological effects. This pleiotropic activity makes NRs ideal molecular targets for the treatment of MASLD [36]. A summary of NR-related drugs currently used in MASLD-targeted therapy is presented in Table 1 (Figure 3).

### 3.1. THR Agonists

#### 3.1.1. Resmetirom

In 2024, the U.S. FDA announced the approval of Rezdiffra (active ingredient is resmetirom), an oral small molecule THR-β agonist, for the treatment of adult MASH patients with moderate to advanced fibrotic disease [16]. This represents the first FDA-approved targeted drug specifically for the treatment of MASLD. In a Phase III clinical trial, resmetirom demonstrated significant efficacy, with 25.9% and 29.9% of patients receiving the 80 mg and 100 mg doses, respectively, achieving resolution of MASH symptoms without worsening of fibrosis at week 52, while only 9.7% in the placebo group [80]. Mechanistically, resmetirom promotes fatty acid catabolism and reduces hepatic lipid accumulation [33]. The approval of Rezdiffra highlights the potential therapeutic benefits of targeting THR-β in the clinical management of MASLD.

#### 3.1.2. MB07811

MB07811 is another THR-β agonist that has been shown to reduce LDL-C and inhibit lipid accumulation in both normal and diet-induced fatty liver rat models. Its effects are mediated by the regulation of lipid metabolism associated genes and downregulation of key lipogenic factors, such as SREBP-1c and apolipoprotein C3 (ApoC3) [81]. Similar results were observed in a phase II trial of MB07811, where its administration significantly reduced LDL-C and liver fat content in patients, and more importantly, MB07811 was found to be safe and well-tolerated [82].

### 3.2. PPAR Agonists

#### 3.2.1. Rosiglitazone

Rosiglitazone, a PPAR-γ agonist, acts as an insulin sensitizer and is primarily used in the treatment of type 2 diabetes mellitus. Its preventive effects on MASLD are attributed to the systemic activation of PPAR-γ, which influences multiple metabolic pathways across various organs, promoting the fat deposition in adipose tissue rather than in the liver, thereby reducing hepatic steatosis [17]. While rosiglitazone also activates PPAR-γ in hepatocytes, promoting hepatic lipogenesis, its activation in peripheral subcutaneous adipose tissue enhances thermogenesis and energy expenditure, and promotes the redistribution of lipids from the liver to adipose depots [88]. Supporting this finding, the deletion of hepatocyte-specific PPAR-γ enhances the therapeutic effect of rosiglitazone in MASLD [89]. In a clinical trial involving 20 patients, rosiglitazone treatment resulted in significant reductions in mean fasting glucose, fasting plasma insulin, alanine aminotransferase (ALT) levels, and the homeostasis model assessments of insulin resistance, indicating improved metabolic and liver function [83]. However, despite these benefits, rosiglitazone has now been withdrawn from the European market due to concerns over increased cardiovascular mortality, one of its major adverse effects [90].

#### 3.2.2. Pioglitazone

Pioglitazone, a thiazolidinedione derivative and PPAR-γ agonist, enhances insulin sensitivity and improves both glucose and lipid metabolism in patients with type 2 diabetes mellitus [18]. Although pioglitazone exhibits weaker PPAR-γ agonism compared to rosiglitazone, it demonstrates superior efficacy in ameliorating MASH [91]. A meta-analysis of pioglitazone revealed significant improvements in liver histologic scores, including reductions in steatosis, inflammation, and ballooning grades; however, pioglitazone did not significantly improve the hepatic fibrosis staging [84].

#### 3.2.3. Dual and Pan PPAR Agonists

Dual and pan PPAR agonists have shown greater promise than subtype-selective PPAR agonists in the treatment of MASLD/MASH [92]. Telmisartan, a dual PPAR-α/γ agonist, not only enhances insulin sensitivity and controls hypertension, but also promotes the expression of genes involved in β-oxidation and fat deposition in adipocytes [22]. Furthermore, telmisartan downregulates genes associated with pro-inflammation and oxidative stress, contributing to its prevention and treatment of MASLD [22].

Saroglitazar, another dual PPAR-α/γ agonist, was approved by the Drug Controller General of India in 2020 for the treatment of MASH [22]. In a clinical trial involving 221 patients, saroglitazar treatment significantly improved lipid profiles, including reductions in total cholesterol, TGs, LDL cholesterol, and VLDL-C [85].

Lanifibranor is a pan PPAR agonist that simultaneously activates all three PPAR-α/δ/γ isoforms. Lanifibranor demonstrated robust efficacy in preventing hepatic steatosis, inflammation, hepatocellular ballooning, and fibrosis in rodent models [93]. In a clinical trial involving 247 patients with MASH, 1200 mg and 800 mg of lanifibranor reduced MASH SAF-A scores and fibrosis progression by 49% and 39%, respectively, and only a 22% reduction in the placebo group [86]. These findings highlight the therapeutic potential of pan-PPAR agonists in addressing multiple pathological features of MASH.

### 3.3. FXR Agonists

#### 3.3.1. Obeticholic Acid (OCA)

OCA is a representative selective agonist of FXR. Bile acids (BAs), the endogenous FXR ligands and the final steroidal products of cholesterol catabolism, are primarily categorized into chenodeoxycholic acid (CDCA) and cholic acid (CA). OCA is a semisynthetic BA derivative, designed using a BA scaffold, and exhibits enhanced potency and efficacy as an FXR agonist. In a phase II clinical trial, administration of OCA at 25 mg/day or 50 mg/day for six consecutive weeks significantly improved insulin resistance, reduced biomarkers of hepatic inflammation and fibrosis, and was well tolerated in patients with MASLD [23].

#### 3.3.2. EDP-305

EDP-305 is a non-BA-derived steroidal FXR agonist that modulates multiple signaling pathways involved in anti-adipogenic, anti-inflammatory, and anti-fibrotic gene expression. In a mouse model of MASH, EDP-305 significantly reduced hepatic adiposity and attenuated fibrosis progression [94]. In a phase II trial, EDP-305 treatment led to reductions in ALT levels and hepatic fat content in patients with MASH, although it dose-dependently increased the incidence of generalized pruritus, a commonly reported adverse effect of FXR agonists [87].

## 4. Conclusions and Perspectives

The pathogenesis of MASLD is complex and multifactorial, involving intricate crosstalk among metabolic dysregulation, chronic inflammation, and fibrotic remodeling. NRs play central roles in regulating a wide range of physiological processes, such as lipid and glucose metabolism, immune response, and fibrosis development, making them attractive molecular targets for the treatment of MASLD. In recent years, considerable progress has been made in the development of drugs that target nuclear receptor signaling pathways, with clinical studies demonstrating promising therapeutic outcomes for the treatment of MASLD.

Although a handful of drugs for MASLD have received FDA approval, several key areas in MASLD drug discovery and development warrant further investigation. First, there is a critical need for therapeutic strategies that enhance efficacy while minimizing adverse effects. Optimizing drug safety profiles remains a central goal in the clinical management of MASLD. Second, given the redundancy and compensatory mechanisms within these complex metabolic, inflammatory, and fibrotic pathways of MASLD, the single-target therapies often exhibit limited effectiveness. Thus, it is essential to explore novel agents capable of modulating multiple NRs or broad-spectrum NR targets to achieve more comprehensive disease control. Third, a deeper understanding of the molecular mechanisms governing NR signaling and regulation is crucial. Advances in these aspects will facilitate the rational design of next-generation therapeutics, especially the artificial intelligence-assisted drug discovery. Fourth, considering the systemic actions of NRs, the development of organ specific therapeutics, targeting tissues such as the liver or subcutaneous adipose tissue, could enhance treatment efficacy while reducing off-target effects. Lastly, in addition to using NR agonists, gene editing techniques such as CRISPR/Cas9 and RNAi can be used to modulate the expression of nuclear receptor genes for treating MASLD.

## Figures and Tables

**Table 1.** Nuclear receptor-related drugs used in MASLD-targeted therapy.

Agent (Trial Name)	Primary Mechanism	Major Inclusion Criteria	Primary Outcome (s)	Refs.
Resmetirom	THR-β agonist	Treatment of adult patients with MASH with moderate to advanced fibrotic disease	Elimination of MASH symptoms without worsening of fibrosis	[80]
MB07811	THR-β agonist	Treatment of MASLD with elevated LDL-C	Reduction in LDL-C and liver fat content	[81,82]
Rosiglitazone	PPAR-γ agonist	Treatment of type 2 diabetes mellitus	Reduction in mean fasting glucose, fasting plasma insulin, and mean ALT	[83]
Pioglitazone	PPAR-γ agonist	Treatment of type 2 diabetes mellitus	Improvement in steatosis grades, inflammation grades, and ballooning grades	[18,84]
Telmisartan	PPAR-α/γ dual agonist	Treatment of patients with hypertension	Improvement in insulin sensitivity	[22]
Saroglitazar	PPAR-α/γ dual agonist	Treatment of patients with noncirrhotic	Improvement in total cholesterol and triglycerides	[85]
Lanifibranor	PPAR pan agonist	Treatment of patients with noncirrhotic, highly active MASH	Reduction in MASH SAF-A scores and fibrosis	[86]
Obeticholic acid	FXR agonists	Treatment of patients with MASH with stage 4 fibrosis	Improvement in IR and reduction in hepatic inflammation and fibrosis	[23]
EDP-305	FXR agonists	Treatment of patients with histologic or phenotypic MASH	Improvement in ALT and hepatic fat content	[87]
